# Association, Distribution, Liberation, and Rheological Balances of Alkyldimethylbenzylammonium Chlorides (C12–C16) †

**DOI:** 10.3390/molecules22101802

**Published:** 2017-10-24

**Authors:** Zuzana Vitková, Jarmila Oremusová, Petra Herdová, Oľga Ivánková, Anton Vitko

**Affiliations:** 1 Department of Galenic Pharmacy, Faculty of Pharmacy, Comenius University in Bratislava, Odbojárov 10, 832 32 Bratislava, Slovak; Herdova@fpharm.uniba.sk; 2 Department of Physical Chemistry of Drugs, Faculty of Pharmacy, Comenius University in Bratislava, Odbojárov 10, 832 32 Bratislava, Slovak; oremusova@fpharm.uniba.sk; 3 Department of Structural Mechanics, Faculty of Civil Engineering, Slovak University of Technology in Bratislava, 810 05 Bratislava, Slovak; olga.ivankova@stuba.sk; 4 Institute of Robotics and Cybernetics, Faculty of Electrical Engineering and Information Technology, 812 19 Bratislava, Slovak; anton.vitko@stuba.sk

**Keywords:** hydrogels, association of surfactants, partition coefficient, release of chlorhexidine, rheological properties

## Abstract

It is known that cationic surfactants have an antimicrobial effect and act as enhancers. This paper studies three cationic surfactants from the group of alkyldimethylbenzylammonium chlorides (dodecyl-, tetradecyl-, and hexadecyl). Interest is focused on the association of the surfactants with respect to temperature, partition balances and their influence on drug release, rheological properties, and the pH of hydrogels. The critical micelle concentrations (CMC) of the surfactants were estimated from dependencies of conductivity, density, spectrofluorimetry, and UV–VIS spectrophotometry on molarity in the temperature range of 25–50 °C. It was found that the temperature dependence of a CMC is U-shaped, with its minimum at 30 °C, and the CMC value decreases as the length of the chain increases. The pseudo-phase separation model was used for the calculation of various thermodynamic parameters, such as the Gibbs free energies (spontaneous process), enthalpies (exothermic process), and entropies of the micelles’ formation, CMCs, and the degree of counterion binding. All thermodynamic parameters, as functions of the temperature, were estimated. It was found that partition coefficients increase as the length of the alkyl chain and the pH = (5.0–7.0) increase. The influences of surfactants, below and above the CMC, on drug (chlorhexidine dihydrochloride) release from hydrogels, rheological properties, and pH at 30 °C were studied. Also, the amounts of the released drug increase as the alkyl chains of the surfactants prolongate. The amounts of the released drug with the surfactant below the CMC are greater than that above the CMC. All hydrogels (regardless of the length of the alkyl chain) exhibit a non-Newtonian pseudo-plastic flow. The results obtained will be used in the formulation of the drug and surfactants into dosage forms.

## 1. Introduction

The optimization of drugs to reach their maximum therapeutic effect is a primordial problem of pharmaceutical technology, and it requires deep knowledge about the influences of auxiliary substances. Appropriate choices of auxiliary substances determine the first process of LADME, namely drug liberation, which in turn influences the subsequent processes, i.e., absorption, distribution, metabolism, and excretion. Therefore, the LADME process should be modelled and analyzed as a whole, which has been a long-lasting research subject of the authors [1,2]. Therefore, in those studies, various roles of surfactants have been studied, which are known to play a vital role not only in the process of LADME, but also in many processes of interest in both the fundamental and applied sciences [3]. One important advantage of surfactants is their ability to formulate oriented colloidal aggregates: micelles were first described by Mc Bain in 1913 [4]. The concentration of a surfactant at which micelles appear is known as the critical micelle concentration (CMC). Its value depends on numerous factors, such as temperature, length of alkyl chain, and influence of counter ions [5].

Numerous papers dealing with the phenomenon of micelle formation have been published. See for instance references [6,7]. The phenomenon has been considered from two primary viewpoints. One considers micelles as chemical species: the mass action model, which has been used ever since the discovery of micelles; while the other considers them as a separate phase: the phase separate model.

The temperature dependencies of ionic surfactants are not linear. That is because the changing temperature initiates a change of interactions between the hydrophilic and hydrophobic parts of a molecule. The values of the CMC first decrease as the temperature increases, and on reaching the minimum they start to increase. Hence, the dependencies are U-shaped curves. That is because two influences act against each other. The increased temperature causes a decrease of the hydrophilic group, what supports the micellization of the surfactant. At the same time, it develops disruptions in the structuralized water around the hydrophilic group, which in turn suppresses micellization. The ratio of the strengths of these effects decides whether the value of the CMC increases or decreases in a given temperature interval [8].

Surfactants are able to increase the solubility of poorly soluble drugs in water, for instance chlorhexidine dihydrochloride (an antiseptic agent with bactericidal, bacteriostatic, and antifungal properties) [9].

Among the physicochemical properties of surfactants, which are influenced by a change in chemical structure, the partition coefficient characterizes lipophility, as it determines the behavior of the substance on the phase interface between the organic solvent and water [10,11].

The aims of the study were as follows.

(1) *Association Balances*

The aim of this part was to determine and compare the CMC values, the degree of counterion bindings, and the thermodynamic parameters of micellization (molar Gibbs energy and the enthalpy and entropy of micelle formation) of three cationic surfactants from the group of alkyldimethylbenzylammonium chlorides where the alkyl was dodecyl: C12, tetradecyl: C14, and hexadecyl: C16. For measurements, four experimental methods were used (conductometry, densitometry, UV–VIS, and spectrofluorimetry). The results obtained were mutually compared and their suitability for the solution of the association balances of the surfactants were evaluated.

(2) *Partition Balances*

The aim of this part was to determine the dependence of the partition coefficient of the studied surfactants on the length of the alkyl chain and the change of pH of the water phase. The partition balance was measured in solution within the pH range of 5.0–7.5 in the system octanol/pH. This interval of pH is interesting and also important for the preparation of hydrogels.

(3) *Liberation Balances*

The aim of this part was to design hydrogels with chlorhexidine as a drug. The permeation characteristics of the drug and rheological properties of the hydrogels were investigated. The drug release profiles of the hydrogels without and with the surfactant were measured, analyzed, and finally compared. Experiments were aimed at the evaluation of the release kinetics contained in the same amounts of the drug (chlorhexidine 0.1% *w*/*w*) with the surfactant, both above and below the CMC.

(4) *Rheological Balances*

The rheological studies confirmed the influence of formulation characteristics on pharmaceutical availability.

## 2. Results and Discussion

### 2.1. Association Properties

#### Critical Micelle Concentration of Surfactants

**Figure 1:**
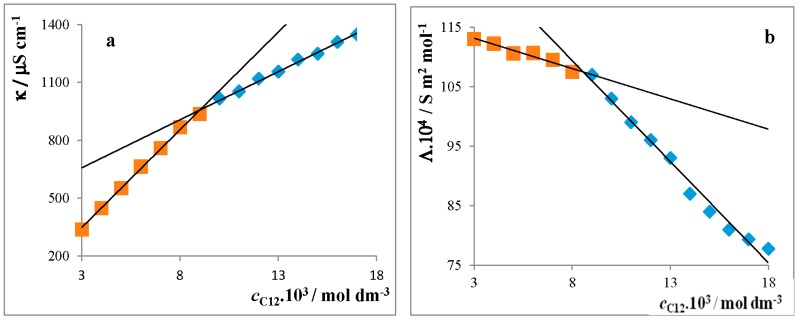

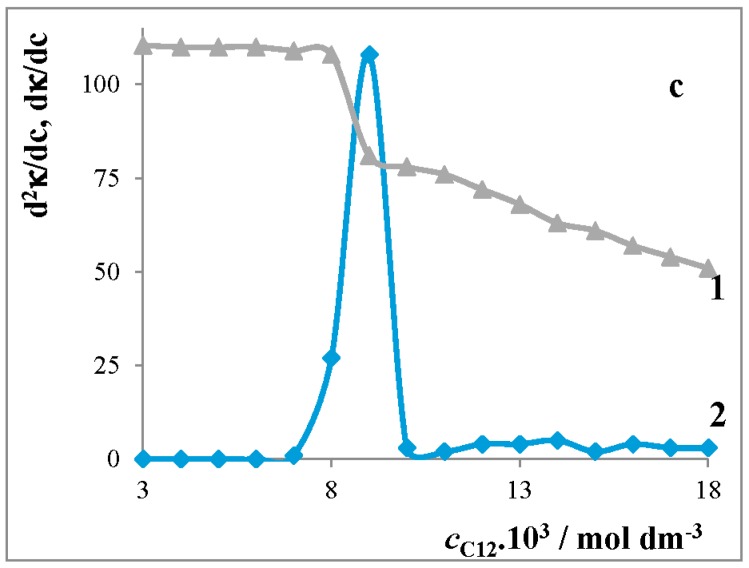
Dependence (**a**) *κ* = *f*(*c*_C12_); (**b**) *Λ* = *f*(*c*_C12_); and (**c**) curve **1**: dκ/dc = *f*(*c*_C12_) and curve **2**: d^2^κ/dc = *f*(*c*_C12_) for surfactant C12 at the temperature 30 °C.

In this part, we studied the effect of both the length of the alkyl chain and temperature on the CMC value, degree of counterion bindings, and the thermodynamic parameters of the micellization of three cationic surfactants (alkyldimethylbenzylammonium chlorides where the alkyl is dodecyl: C12, tetradecyl: C14, and hexadecyl: C16). Four experimental methods—conductometry, UV–VIS spectrophotometry, spectrofluorimetry, and densitometry—were used. The critical micelle concentration of ionic surfactants is usually determined by the intersection of two straight lines on plots of the conductivity (*κ*) or molar conductivity (*Λ*) versus the concentration of surfactants: molarity (*c*), molality (*b*), or molar fraction (*x*). For example, the conductivity measurement of the substance C12 in the solution (conductivity *κ* versus molarity: Figure 1a, and molar conductivity *Λ* versus molarity: Figure 1b) at 30 °C. Curves are drawn for only one of the three measurements of the same surfactant: C12.

For the determination of precise values of the CMC, Mata et al. [12] suggested using the first or second derivation of the plots on conductivity (d*κ*/d*c*) or (d^2^*κ*/d*c*^2^) versus molarity. The inflection point of the sigmoid curve (Figure 1c, curve 1) gives the CMC value determined from the first derivation. In the case when the second derivation was used, the CMC was determined from the maximum point of curve (Figure 1c, curve 2). The CMC values calculated from conductometric measurements by various procedures showed good correspondence. Mehta [13] studied the dependence of a CMC on the chemical structure of the selected surfactants and made some general remarks, namely that the CMC decreases strongly with an increasing length of the alkyl chain of the surfactants. Our experimental results certify that assertion: Table 1.

**Figure 2:**
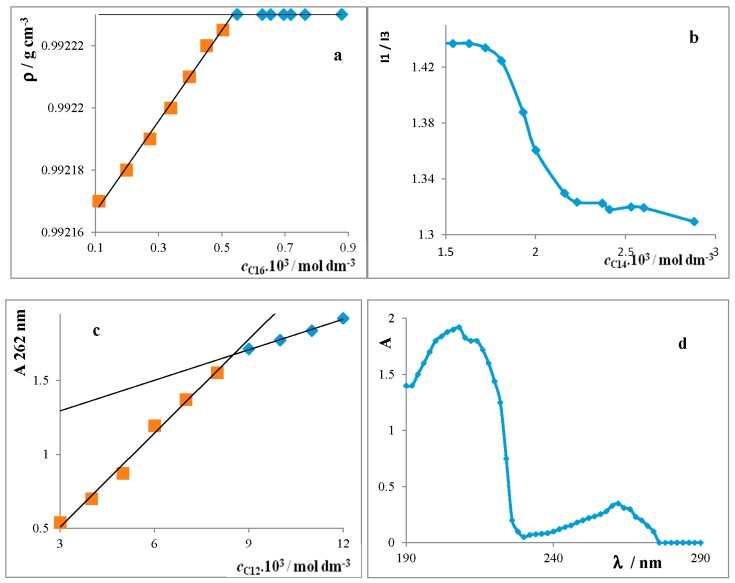
Dependence of *ρ* = *f*(*c*_C16_): (**a**) (*t* = 40 °C); I_1_/I_3_ = *f*(*c*_C14_); (**b**) (*t* = 25 °C); *A* = *f*(*c*_C12_); (**c**) (*t* = 25 °C), and (**d**) absorption spectrum C12.

For reliable verification, another three experimental methods of determination for the CMC were used: densitometry (CMC measured in the temperature range of 20–50 °C), Figure 2a; spectrofluorimetry; and spectrophotometry (25 °C), Figure 2b–d and Table 1. It will be shown that all three methods give comparable results.

The micellization and CMC of ionic surfactants are affected by various factors, including temperature, as the hydrophobic and hydrophilic interactions depend on the temperature. Studies of the CMC versus temperature have been performed to obtain information on these interactions. For the sake of better visibility, shown are various CMC values which were determined at different temperatures for all studied surfactants, see Table 1.

The CMC values of all studied surfactants pass at the temperature 30 °C through a shallow minimum. Their CMC values with respect to the length of the alkyl chain are ordered as follows:

C12 > C14 > C16.

It is known that at a higher temperature the thermal motion increases, which results in de-micellization owing to the distraction of the palisade layer of the micelle, which subsequently enhances the CMC of surfactants [14]. This is because of the facts that at increasing temperature the high solubility of the hydrocarbon stabilizes surfactant monomers, and micelle formation is hindered. This fact results in a higher CMC for all three surfactants [3,15]. Increasing the temperature above 30 °C disfavours micellization, and thereby the increase of the CMC values of the surfactants is explained.

All of the three experimental methods are suitable for an evaluation of the CMC of the surfactants, as there exists good agreement between them (Table 1). Because the studied surfactants dissociate in solutions, we used conductometric measurements. UV–VIS measurements were used due to the existence of significant strips with a maximum at *λ* = 262 nm (Figure 2). The dependencies ρ = *f*(*c*_surf_) show an abrupt change of the line slopes above the CMC (Figure 2a), which justifies using densitometry for the evaluation of the CMC. Pyrene was used in spectrofluorimetry in the role of a probe. The CMC value was determined from the point of inflection of the curve I1/I3 = *f*(*c*_surf_) (Figure 2b).

**Figure 3:**
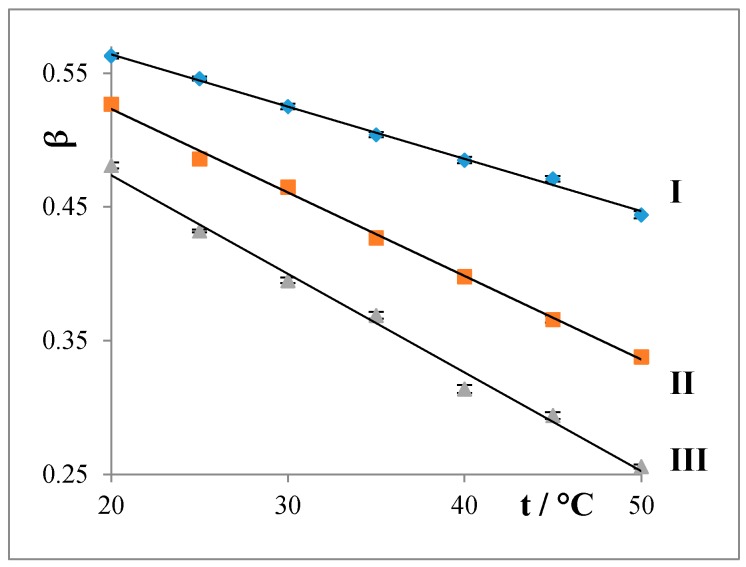
Dependencies of degrees of counterion bindings (*β*) on length of alkyl chain and temperature for the studied surfactants **I**: C12, **II**: C14, and **III**: C16, by using conductometry.

The degrees of counterion bindings (*β*) were calculated from conductivity measurements according to Equation (1); see Figure 3.

The degrees of counterion bindings decrease as the alkyl chain and temperature of the surfactant increases.

**Figure 4:**
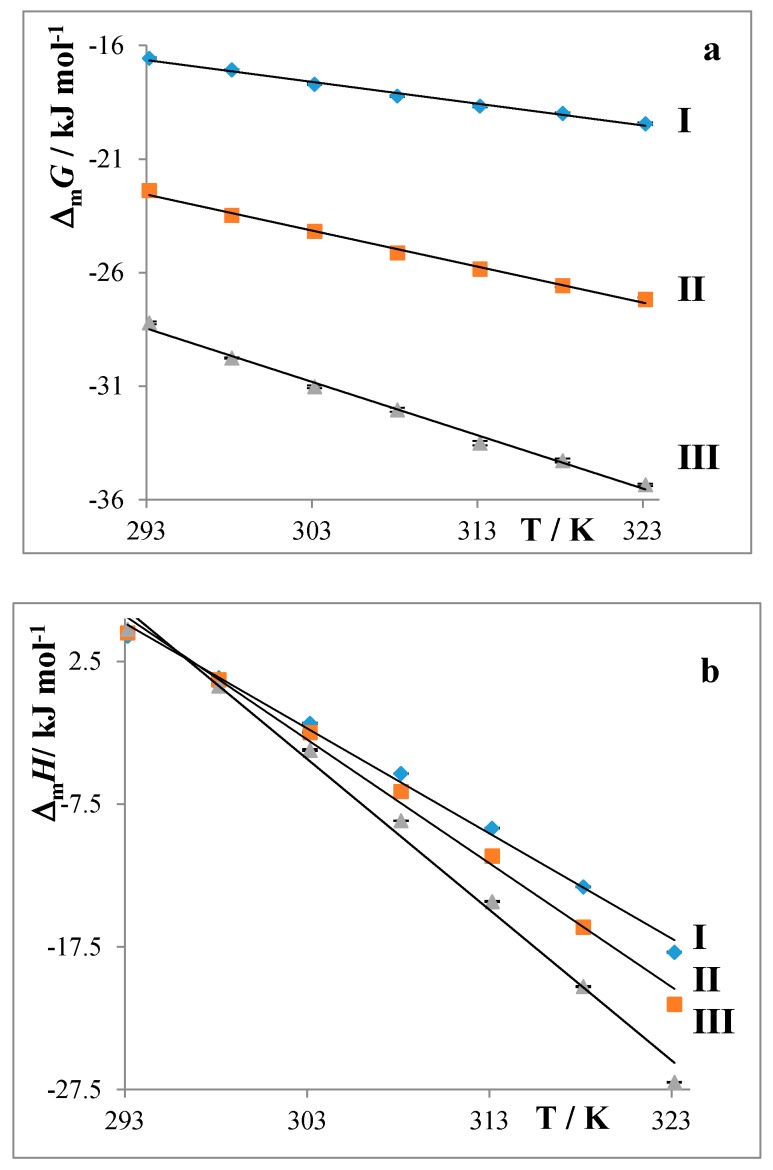

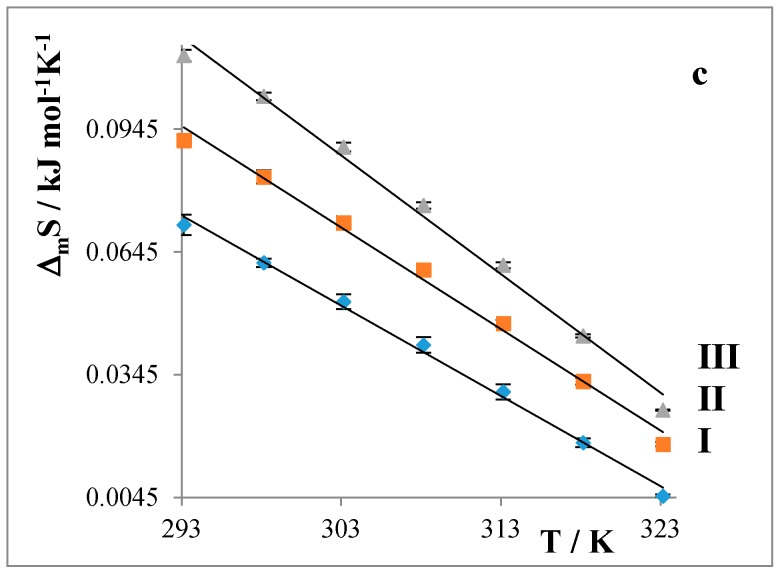
Temperature dependencies of thermodynamic parameters of micellization Δ_m_*G* (**a**); Δ_m_*H* (**b**); and Δ_m_*S* (**c**); surfactants **I**: C12, **II**: C14, and **III**: C16.

The thermodynamic parameters of micellization play a vital role in understanding the process of micellization. The elucidation of these parameters (the molar Gibbs energy Δ_m_*G* and the enthalpy Δ_m_*H* and entropy Δ_m_*S* of micellization) is essential for observations of the effect of structural and environmental factors on CMC values. Experimental values of CMC and degrees of counterion bindings were used for the calculation of thermodynamical parameters describing the micellization process according to Equations (2)–(5). The calculated values are summarized in Figure 4.

It was found that the formation of micelles is connected with negative molar Gibbs energy, i.e., the process of micellization is thermodynamically favoured and spontaneous. Values of Δ_m_*G* decrease with the length of the alkyl chain of the surfactants and also at all studied temperatures (Figure 4a). Data shown in Figure 4b illustrate that the values of Δ_m_*H* highly depend on the temperature and depend less on the alkyl chain. They decrease with increasing temperature and the prolongation of the alkyl chain of the surfactants. The decrease of Δ_m_*H* shows that the process of micellization is becoming more exothermic. The change of Δ_m_*H* may be caused by the sum of two contributions of the opposite sign: the removal of the medium is endothermic and the transfer into the micelle is exothermic.

For the data shown in Figure 4c, the values of Δ_m_*S* are all positive, which indicate that the major driving forces of micellization are hydrophobic interactions. Water molecules in hydration shells around the hydrophobic part of the monomeric amphiphiles are released during micellization. The values are positive within the whole temperature range and they decrease with increasing temperature.

### 2.2. Partition Balances

**Figure 5:**
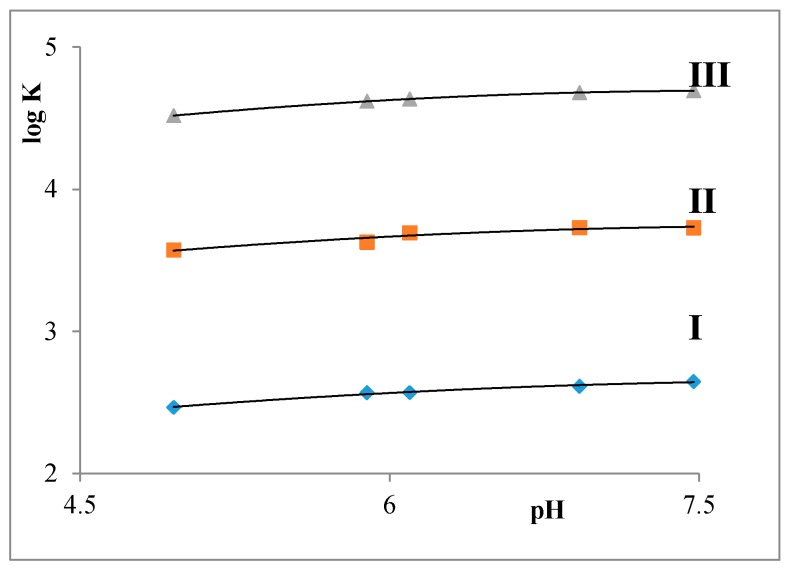
Dependence of partition coefficient of surfactants on pH. **I**: C12, **II**: C14, and **III**: C16.

This part was devoted to the study of the influence of both the length of alkyl chain and pH in the range of 5.0–7.0 on the partition coefficient of the surfactants. Chitosan hydrogels appear around pH = 6–7. Values of log K were obtained as averages of three measurements and they are summarized in Figure 5.

The values of the partition coefficients of all three surfactants increase in the order C12 < C14 < C16 as the pH value of the water phase increases, which corresponds with an increasing lipophility of the surfactants.

### 2.3. Liberation Balances

**Figure 6:**
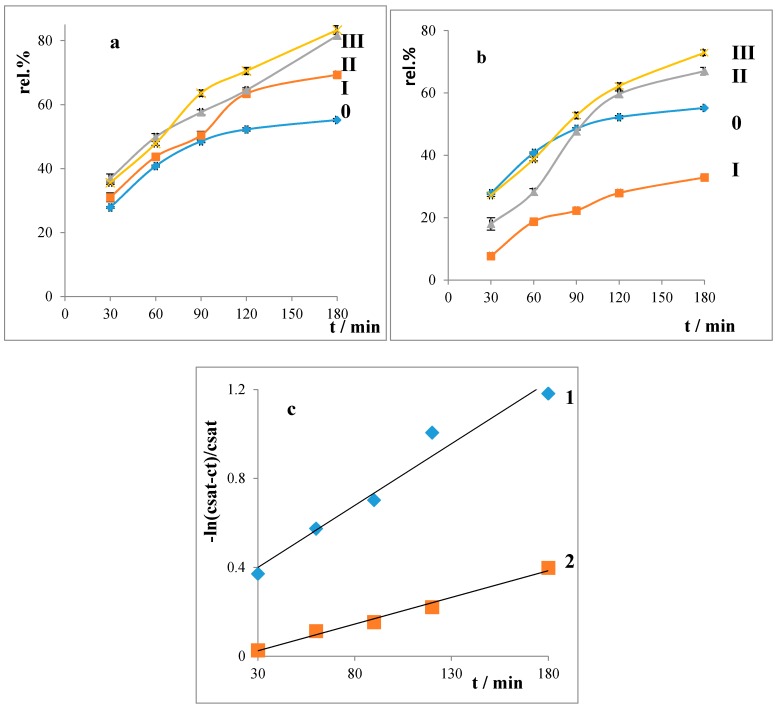
Liberation of CHX (**a**) below the CMC; and (**b**) above the CMC of surfactants, numbers: curve release of CHX from hydrogel without surfactants, **I**: C12, **II**: C14 and **III**: C16; (**c**) curves for the determination of the release rate constants of CHX from hydrogels with surfactant C12 **1**-below CMC and **2**-above CMC pri *t* = 30 °C.

The object of this study was drug (chlorhexidine) release from the hydrogels with and without surfactants, and also their concentration above and below the CMC. The aim was to find the influence of the length of alkyl chain on the drug release from the hydrogels. The average values of the released drug are summarized in Figure 6.

From Figure 6, it follows that amount of the released drug is higher for hydrogels with a surfactant below the CMC. That could be explained by the monomer form of the surfactant in the solutions. If the surfactant’s concentration in the hydrogels are above the CMC, the drug is incorporated into micelles, hence it releases slowly. The amount of the released drug increases with the prolongation of the alkyl chain of the surfactant. From the obtained release curves were determined the release rate constants of the pseudo-first order kinetics in accordance with [16]. The obtained values are summarized in Table 2.

It can be seen that all drug release rate constants from the hydrogels with content of surfactant below the CMC are higher, which can be explained by the fact that in these systems the surfactants are present in a monomer form only; hence, they do not influence the release process to such an extent as if they were above the CMC.

**Figure 7:**
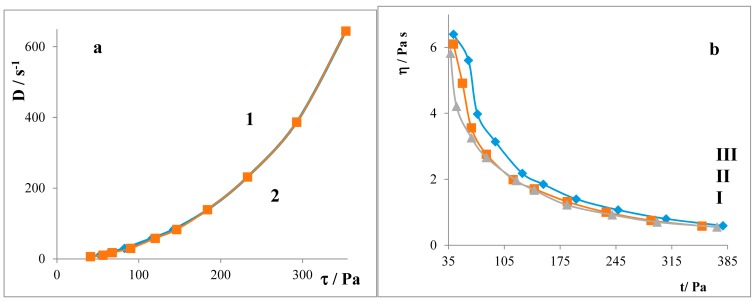
(**a**) Rheogram of hydrogel with C12 below the CMC: curve **1**-increasing, **2**-decreasing (**b**) Dependencies *η* = *f*(*τ*) curve **I**: C12, **II**: C14, and **III**: C16.

From Figure 7, it follows that the flow curves do not exhibit significant differences. It means that the flow is pseudo-plastic.

Table 3 shows pH of the prepared hydrogels and blank.

## 3. Materials and Methods

### 3.1. Drug

Chlorhexidine dihydrochloride (Ph.Eur.9)—(abreviation CHX)—1,1′-(Hexane-1,6-diyl)bis [5-(4-chlorophenyl)biguanide]dihydrochloride, (*M*_r_ = 578.4) was obtained from Imperial Chemical Industries, Geshire, UK.

### 3.2. Auxiliary Substance

**Figure 8:**
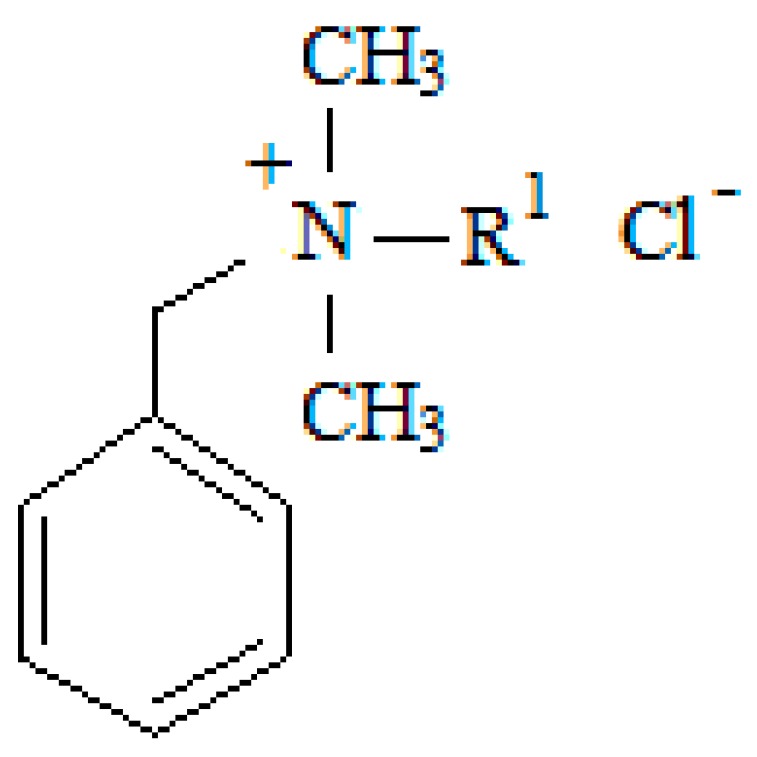
Structural formula of the studied surfactants.

The systems with three surfactants from the group of quaternary ammonium salts having a common benzyldimethylalkylammonium cation and chloride anion were studied (Figure 8). The basic characteristics of the surfactants are summarized in Table 4.

The surfactants were used without further purification.

- As a solvent there was used redistilled water with conductivity *κ* <1.5 μS cm^−1^.
- Pyrene (abbreviation Pyr)—fenantrene—*M*_r_ = 202.25 and density *ρ* = 1.271 g mol^−1^ (Merck, Darmstadt, Germany).
- Universal buffer, 1000 mL of it contains 0.04 mol dm^−3^ H_3_PO_4_ (4.9 g 80% dm^−3^ H_3_PO_4_), 0.04 mol dm^−3^ CH_3_COOH (2.4 g CH_3_COOH), 0.04 mol dm^−3^ H_3_BO_3_ (2.474 g H_3_BO_3_), was mixed with a mL 0.2 mol dm^−3^ of solution of NaOH. The value of pH was within the interval 11.98–2.09. All used chemicals were of purity p.a.
- Chitosan—medium (abbreviation CHIT)—*M*_r_ = 190.000–375.000) was obtained from Sigma Aldrich Chemie GmbH, Steinheim, Germany.
- Lactic acid solution (abbreviation LA) was supplied by Merck Chemical Company, (Germany).
- The 1-octanol for UV–VIS was supplied by Merck Chemical Company, (Germany).

## 4. Methods and Computational Procedures

### 4.1. Determination of the CMC Value

#### 4.1.1. Conductivity

Conductivity dependencies were measured by conductometric titration (by dilution of more concentrated solution with redistilled water) at the temperature range of 20–50 °C using a digital conductomer ino Lab (Germany), with the double conductivity cell and platinum electrode Tetra Con 325 (cell constant K = 0.474 μS cm^−1^). The precision of the measurements was ± 0.01 μS cm^−1^. The solutions were continually stirred and thermostated (thermostat JULABO 5E: Swiss) of the precision ± 0.1 °C. Values of CMC were estimated from measured dependencies (*κ* = *f*(*c*_surf_), *Λ* = *f*(*c*_surf_), d*κ*/d*c* = *f*(*c*_surf_), or d^2^*κ*/d*c* = *f*(*c*_surf_)).

#### 4.1.2. Degree of Counterion Binding (*β*)

Values of *β* were determined from the slopes of two linear parts of the conductivity curves (*κ* = *f*(*c*_surf_)) and calculated according Equation (1):

(1) $$\beta =1-\frac{S_{2}}{S_{1}}$$

where *S*_1_ is the slope of the linear parts of the conductivity curve below the CMC, and *S*_2_ is the slope above the CMC. Units of *S*_1_ and *S*_2_ are *S* m^2^ mol^−1^ [13].

#### 4.1.3. Thermodynamic Parameters of Micellization

The pseudo-phase model was applied to estimate thermodynamic parameters because it is of wide acceptance for the interpretation of the energetic state of micelle formation.

The CMC and *β* values were used in the calculation of the thermodynamic parameters of micellization according to the equations [17]:

Molar Gibbs energy of micellization (*Δ_m_G*):

(2) $$\Delta_{m}G=(2-\beta )RT\ln CMC\;(J\;{\mathrm{mol}}^{-1}).$$

Molar enthalpy of micellization (*Δ_m_H*):

(3) $$\Delta_{m}H=-(2-\beta )RT^{2}\frac{\partial (\ln CMC)}{\partial T}=-(2-\beta )RT^{2}(B+2CT)\;(J\;{\mathrm{mol}}^{-1})$$

where *B* and *C* are the parameters of the second-order polynomial:

(4) $$\ln CMC=f(T)=A+BT+CT^{2}.$$

Molar entropy of micellization (*Δ_m_S*):

(5) $$\Delta_{m}S=\frac{\Delta_{m}H-\Delta_{m}G}{T}\;(J\;{\mathrm{mol}}^{-1}\;K^{-1}).$$

#### 4.1.4. Spectrophotometry

The absorbance (*A*) of the solutions for association and partition were measured with a UV–VIS spectrophotometer Hewlett Packard 8452A (diode array) in 0.5 or 1.0 cm cuvettes at *λ*_max_ = 262 nm and a temperature of 25 °C. As a blank was used distilled water. The absorbance (*A*) of the solutions of hydrogels was measured in 1.0 cm cuvettes at *λ*_max_ = 254 nm and a temperature of 30 °C.

#### 4.1.5. Densitometry

The samples were prepared by an accurate weighting of the surfactants and redistilled water. All prepared solutions were degassed (10 min) before the densitometry measurement. Density was measured by the densitometer M 4500, Anton Paar (Vienna, Austria) at the temperature range of 20–50 °C. The CMC values of the surfactants were calculated from the dependence (*ρ* = *f*(*c*_surf_)) at the different temperatures.

#### 4.1.6. Spectrofluorimetry

Into the prepared solutions of surfactants was (before the measurement) added 6 μL of pyrene (*c*pyr = 5 × 10^−4^ mol dm^−3^) used in the role of a fluorimetric probe. The peaks of intensities *I*_1_ and *I*_3_ were measured with the spectrofluorimeter Fluoromax-4 Horiba Jobin Yvon (Edison, NJ, USA) under the following conditions: excitation wave length = 332 nm, excitation slot = 2 nm, emission spectrum = 340–450 nm, step size = 1 nm, and emission slot = 2 nm. From the dependence *I*_1_/*I*_3_ = *f*(*c*_surf_) sigmoidal curve was calculated the CMC values at the temperatures 25–50 °C.

#### 4.1.7. Determination of Partition Coefficients

The experimental partition coefficient was determined by the method “shake-flask” between phase o/w. In the role of the water phase (*w*) was used solutions of different pH (universal buffer solution) and as a lipophilic phase was used 1-octanol. Masses of surfactants (cca 5 mg) were dissolved in water phase (10 mL) and octanol (5 mL). Solutions were intensively shacked for 1 h, and after 24 h of staying the absorbance was measured at the wavelength 262 nm. The absorbance was recalculated by calibration curves at concentrations and the values of partition coefficients were calculated in accordance with the equation:

(6) $$K=\frac{m_{0}-c_{W}MV_{W}}{c_{W}MV_{O}}$$

where:

*m*_0_: mass of sample (surfactants) (kg).
*c*_W_: molarity of surfactant in water phase (mol m^−3^).
*M*: molar mass of surfactant (kg mol^−1^).
*V_W_*: volume of water phase (m^3^).
*V_O_*: volume of octanol phase (m^3^).

#### 4.1.8. Preparation of Hydrogels

Chitosan hydrogels of concentration 2.5% (*w*/*w*) were prepared in 1% (*w*/*w*) lactic acid solution with and without surfactants. The concentration of chlorhexidine was 0.1% (*w*/*w*). Gels of chlorhexidine were prepared with the three surfactants C12–C16 (Table 4). The concentrations of surfactants were above and below their CMC values. The total mass of the hydrogel was 50 g. The compositions of the hydrogels are stated in Table 5.

#### 4.1.9. Preparation of Dosage Form

The hydrogels were prepared in two parallel samples. Accurate amounts of the gelling agent: chitosan, and the permeation enhancer: surfactant were given into the balanced flask. Then, the appropriate amount of the 1.0% (*w*/*w*) solution of lactic acid was added (up to 50.0 g including other substances). The system was slowly stirred while all particles swelled and dissolved. In this way, the homogenous hydrogel was prepared. The blanks (hydrogels without the drug but with surfactants) were prepared by the same procedure.

The drug was sieved by a sieve with pores of 125 μm to form a very fine powder. At the end, an accurate amount of the drug was gradually added into the prepared gel while the system was continually stirred. The samples were prepared at the laboratory temperature *t* = 25 ± 0.2 °C. For the sake of homogenization of the inner structure, the hydrogels were stored at 5 °C for 48 h.

#### 4.1.10. In Vitro Release

A series of eight Franz diffusion cells was used and the drug release from the hydrogels was evaluated by using a semipermeable membrane. The donor compartment was filled with 0.5 g of hydrogels. The acceptor compartment contained 38 mL of purified water maintained at 30 °C and stirred by a magnetic stirrer. The amounts of the released drug were determined by a UV–VIS spectrophotometer at λ_max_ = 262 nm after 30, 60, 90, 120, 150, and 180 min. The results were evaluated on the basis of the released cumulative amounts of the drug and represent the average of eight measurements. The measurements were performed in two parallel systems. The released amounts were determined in the 48 h after the hydrogels were prepared. The drug concentration of CHX in the solution was calculated for every sample using the specific absorption coefficient $\left(A_{1\mathrm{cm}}^{1\%}=281.81\right)$.

### 4.2. Drug Release Rate Constants

The calculations of the rate constant of the drug chlorhexidine followed from the fact that the drug released in tiny amounts into the water. The release rate constant was calculated according the following equation [16]:

(7) $$-\ln\frac{W_{sat}-W_{t}}{W_{sat}}=kt\;\min^{-1}$$

where: *W_t_* is the concentration of released drug in time *t*; W*_sat_* is the saturated concentration, i.e., the concentration caused by the whole amount of the released drug from the hydrogel; *K* is a release rate constant.

#### Measurement of pH

The pH values of hydrogels were measured by a combined glass electrode of pH-meter Metrohm (WTW series Inolab at 30 °C after 48 h since the preparation).

## 5. Conclusions

The association and partition balances of three cationic surfactants from the group of benzyldimethylalkylammonium chlorides (with alkyl chain C12-dodecyl, C14-tetradecyl, and C16-hexadecyl) were studied.

In particular, association balances were studied in the temperature range of 20–50 °C. From the obtained conductivity curves were calculated the values of CMC, *β*, and thermodynamic parameters of micellization.

### The Conclusions Derived from Association Measurements

- The values of CMC increase with an increasing temperature of the micellization process. The curves of CMC = *f*(*t*) reach a shallow minimum at a temperature of about 30 °C. The values of CMC of the studied surfactants are decrease in the order C12 > C14 > C16, which is caused by increasing lipophility, i.e., the prolongation of the alkyl chain in the molecule of the surfactant.
- For verification of the determined CMC from conductivity measurements were used another three experimental methods: densitometry, spectrofluorimetry, and UV–VIS spectrophotometry. All methods are suitable for the study of the formation of micelles.
- The degrees of counterion bindings (*β*) of the studied water systems linearly decrease with both increasing temperature and prolongation of the alkyl chain of the surfactant.
- For the thermodynamic parameters of micellization:

The values of Δ_m_*G* decrease with the lengths of the alkyl chain of the surfactants as well as the temperature. It was found that the formation of micelles is connected with negative molar Gibbs energy, i.e., the process of micellization is thermodynamically favored and spontaneous.

The values of Δ_m_*H* decrease with increasing temperature as well as with the prolongation of the alkyl chain, which indicates that the process of micellization is becoming more exothermic. All values of Δ_m_*S* are positive, which indicates that the major driving forces of micellization are hydrophobic interactions.

- Partition balances

The values of partition coefficients of all three surfactants are increasing with increasing pH (pH = 5.0–7.5) of the water phase in the order C12 < C14 < C16, which is in correspondence with increasing their lipophility.

- Liberation balances

The amount of the released chlorhexidine dihydrochloride (CHX) increased with the prolongation of the alkyl chain of the surfactants. If the surfactant was below the CMC, a greater amount of the drug permeated through the semipermeable membrane in comparison with the case when the surfactant was above the CMC. That was because, in the case where the surfactant was above the CMC, the drug was closed in the micelles. The release rate constants of the drug from hydrogels are higher in the systems with surfactant concentration below the CMC. All evaluated hydrogels (regardless of the length of the alkyl chain) exhibit a non-Newtonian pseudo-plastic flow. The hydrogels’ pH was not statistically significantly influenced by changing the length of the alkyl chain (average value is pH = 6).

The results obtained are significant for pharmaceutical technology. They will be effectively utilized in the process of drug formulation, as the surfactants are important pharmaceutical auxiliary substances used as enhancers or antimicrobial agents. The found behaviors and relations between parameters such as association, distribution, liberation, and rheological balance provide a drug designer with a serious knowledge base. The obtained knowledge is inevitable for drug optimization because obtaining good release kinetics is necessary for reaching a maximum therapeutic effect. Therefore, the obtained results will be used in ongoing research activities at the workplace of the authors. The aim of this research is to develop optimal control of drug liberation from semisolid dosage forms and computer simulation of the LADME process with possible prediction of the therapeutic effect [1,18,19,20].

## Figures and Tables

**Table 1:** Dependencies of critical micelle concentration (CMC) on the length of the alkyl chain and temperature of the studied surfactants, C12, C14, and C16, by using experimental methods, namely conductometry (*κ*) and densitometry (*ρ*) (20–50 °C), spectrophotometry (A), and spectrofluorimetry (I1/I3) (25 °C).

CMC.10^3^/mol dm^−3^						
Surfactant	*t*/°C	Methode				(CMC ± s_CMC_).10^3^/mol dm^−3^
		*κ* *^1^	*ρ* *^2^	A *^3^	I1/I3 *^4^	
C12	20	8.84	8.83	8.83	8.86	8.837 ± 0.014
	25	8.77	8.71			8.740 ± 0.032
	30	8.55	8.65			8.600 ± 0.059
	35	8.61	8.70			8.655 ± 0.045
	40	8.81	8.90			8.855 ± 0.045
	45	9.14	9.22			9.180 ± 0.040
	50	9.65	9.84		9.62	9.703 ± 0.118
C14	20	1.96	1.98	1.95	1.99	1.970 ± 0.009
	25	1.94	1.96			1.950 ± 0.010
	30	1.92	1.91			1.915 ± 0.005
	35	1.96	1.97			1.965 ± 0.005
	40	2.04	2.04			2.040 ± 0.000
	45	2.14	2.16			2.150 ± 0.010
	50	2.27	2.33		2.25	2.283 ± 0.042
C16	20	0.49	0.50	0.48	0.50	0.493 ± 0.010
	25	0.47	0.47			0.473 ± 0.000
	30	0.46	0.47			0.468 ± 0.005
	35	0.47	0.47			0.472 ± 0.005
	40	0.48	0.48			0.486 ± 0.003
	45	0.50	0.50			0.515 ± 0.001
	50	0.53	0.54		0.54	0.539 ± 0.008

*^1^: unit μS cm^−1^; *^2^: g cm^−3^; *^3^: UV–VIS; *^4^: spectrofluorimetry and standard deviation sCMC.

**Table 2:** Release rate constants of CHX from hydrogels.

Sample	Hydrogel	*k*.10^3^/min^−1^
1	without surfactants	6.90 ± 0.29
2	C12 below CMC	9.09 ± 0.33
3	C12 above CMC	6.70 ± 0.33
4	C14 below CMC	7.99 ± 0.41
5	C14 above CMC	7.69 ± 0.51
6	C16 below CMC	5.56 ± 0.62
7	C16 above CMC	2.40 ± 0.16

**Table 3:** pH of chitosan hydrogels.

Sample	Hydrogels	pH	
		Blank	Hydrogel
1	without surfactant	6.25	6.06
2	C12 below CMC	6.20	6.13
3	C12 above CMC	6.16	6.05
4	C14 below CMC	6.03	6.06
5	C14 above CMC	6.00	6.02
6	C16 below CMC	5.98	6.03
7	C16 above CMC	6.09	6.35

**Table 4:** Characteristics of studied alkylbenzyldimethylammonium chloride.

Surfactant	Formula	*M*_r_	m.p. (°C)	R^1^	Purity (%)	Supplier
C12	C_21_H_38_NCl	339.99	60–61	dodecyl-(CH_2_)_11_-CH_3_	99	FLUKA
C14	C_23_H_42_NCl	368.05	53–56	tetradecyl-(CH_2_)_13_-CH_3_	99	FLUKA
C16	C_25_H_46_rNCl	396.10	55–69	hexadecyl-(CH_2_)_15_-CH_3_	97	FLUKA

**Table 5:** Composition of prepared hydrogels.

% (*w*/*w*)				*m*_surf_/g		
Sample	CHIT	LA	CHX	C12	C14	C16
1	2.5	1.0	0.1	-	-	-
2	2.5	1.0	0.1	0.015	-	-
3	2.5	1.0	0.1	0.222	-	-
4	2.5	1.0	0.1	-	0.004	-
6	2.5	1.0	0.1	-	0.361	-
6	2.5	1.0	0.1	-	-	0.010
7	2.5	1.0	0.1	-	-	0.015

CHIT: chitosan; LA: lactic acid.
